# Online-Befragung der Ärzteschaft zu Kenntnissen über Berufskrankheiten

**DOI:** 10.1007/s40664-022-00475-9

**Published:** 2022-07-26

**Authors:** Beatrice Thielmann, Irina Böckelmann

**Affiliations:** Bereich Arbeitsmedizin, Medizinische Fakultät, Otto-von-Guericke-Universität Magdeburg, Leipziger Str. 44, 39120 Magdeburg, Deutschland

**Keywords:** Qualitätssicherung der Lehre, Relevanz, Berufskrankheiten, Berufsbedingte Krebserkrankungen, Fortbildung, Teaching quality assurance, Relevance, Occupational diseases, Occupational cancers, Training

## Abstract

**Hintergrund:**

Basiswissen über Berufskrankheiten (BK) ist für jeden Arzt erforderlich. BK dürfen nicht übersehen werden. Hauptziel dieser Studie war es, das selbsteingeschätzte Wissen zum Thema arbeitsbedingte Erkrankungen (aE) und BK sowie das Handeln bei begründetem Verdacht auf eine BK bei Ärzten verschiedener Fachgebiete zu erfassen und Ärzte verschiedener Fachrichtungen in Bezug auf dieses Thema zu sensibilisieren, insbesondere für die berufsbedingten Krebserkrankungen.

**Methodik:**

Im Zeitraum von 11/2014 bis 5/2015 erfolgte eine Online-Befragung unter Ärzten verschiedener Fachrichtungen.

**Ergebnisse:**

Es nahmen 254 in Sachsen-Anhalt registrierte Ärzte/innen, die ihr Studium in verschiedenen Bundesländern absolviert haben, an der Befragung teil. Der größte Anteil der Ärzte/innen (69,7 %) war zwischen 40 und 59 Jahre alt. Der überwiegende Teil der Ärzteschaft verfügte über 16 bis 30 Berufsjahre. Knapp ein Viertel aller Befragten schätzte den eigenen Kenntnisstand zu diesem Thema als mangelhaft/ungenügend ein. Die Hälfte der Befragten gab eine nicht ausreichende Vorbereitung zu dieser Problematik, während des Studiums oder während der Facharztausbildung, in ihrem Fach an. 91,1 % der Befragten unterschätzten die Wichtigkeit der arbeitsmedizinischen Kenntnisse auf diesem Gebiet während des Studiums teilweise oder komplett. Knapp drei Viertel der Befragten haben während ihrer beruflichen Tätigkeit noch keine Fortbildungsangebote zu dieser Thematik wahrgenommen.

**Diskussion:**

Es bestehen hohe fachliche und ethische Anforderungen an den Arzt, BK frühzeitig zu erkennen und der Anzeigepflicht bei BK nachzugehen. Die Befragung diente u. a. auch dem Ziel, die Wege der Qualitätssicherung der universitären Lehre sowie von Fort- und Weiterbildungen mit einem interdisziplinären Charakter aufzuzeigen. Gemeinsame Fortbildungen zur Vermittlung der arbeitsmedizinischen Kompetenz zum Thema Berufskrankheiten sollten vermehrt angeboten werden. Die Kommunikation in der Lehre soll aufzeigen, wie wichtig die arbeitsmedizinischen Kenntnisse für jeden Arzt sind.

## Abstract (en)

**Background:**

Basic knowledge of occupational diseases is necessary for every physician. Occupational diseases must not be neglected. The aim of this survey was to sensitize physicians from various specialties regarding the topic of work-related illnesses and occupational diseases. The knowledge on this topic was to be evaluated. Furthermore, this online survey should be used for quality management of occupational medicine teaching as well as further education and training.

**Methods:**

In the period from 11/2014 to 5/2015, an online survey was conducted.

**Results:**

A total of 254 people answered the survey, which was registered in Saxony-Anhalt and 69.7% of the physicians were between 40–59 years old. Almost 1/4 of all respondents assessed their own level of knowledge on this topic as poor/insufficient. Half of the respondents reported inadequate/insufficient training on this topic during their studies or during residency training in their specialty. Of the respondents 91.1% partially or completely underestimated the importance of occupational health knowledge on this topic during their studies. Almost ¾ of the respondents had not yet taken advantage of any training courses on this topic during their professional activity.

**Conclusion:**

There are high professional and ethical requirements for physicians to recognize and report occupational diseases. Further training in occupational medicine on this subject should be offered more frequently and the presence of occupational medicine in medical studies at universities should be increased.

Ausreichendes Wissen der Ärzteschaft^1^ über mögliche Zusammenhänge von beruflicher Tätigkeit und einer Erkrankung ihres Patienten ist eine der wichtigsten Grundlagen für die Entscheidung der Erstellung einer Anzeige über den begründeten Verdacht einer Berufskrankheit (BK). „Berufskrankheit“ ist ein Rechtsbegriff, kein medizinischer Terminus. „Berufskrankheiten sind Erkrankungen, die Versicherte durch ihre berufliche Tätigkeit erleiden und die in der Berufskrankheiten-Verordnung (BKV) aufgeführt sind“ [1]. Das sind Krankheiten, die die Bundesregierung aufgrund von § 9 Abs. 1 SGB VII durch Rechtsverordnung mit Zustimmung des Bundesrates als BK bezeichnet [2].

Jeder Arzt und Zahnarzt ist nach § 202 SGB VII verpflichtet, den begründeten Verdacht auf das Vorliegen einer Berufskrankheit bei dem zuständigen Träger der Gesetzlichen Unfallversicherung (Berufsgenossenschaft, Unfallkasse) oder alternativ, falls nicht bekannt, dem Staatlichen Gewerbearzt bzw. Landesgewerbearzt anzuzeigen [3]. Das gilt vor allem dann, wenn diese in der Anlage der BK-Verordnung gelistet ist [4]. Neben den Ärzten besteht für Unternehmer Anzeigepflicht, wenn die Anhaltspunkte für eine mögliche BK vorliegen. Des Weiteren können auch Versicherte, Krankenkassen, Rentenversicherungsträger, Arbeitsamt u. a. den Verdacht auf das Vorliegen einer BK anzeigen. Dafür gibt es ein amtliches Formular, welches auf den Seiten der Deutschen Gesetzlichen Unfallversicherung heruntergeladen werden kann [5].

Anerkannte Berufskrankheiten zeigen ein Plus von 104,79 % im Vergleich 2019/2020. Todesfälle infolge einer BK verzeichneten ein Minus von 6,9 % (2019: 2555 Fälle, 2020: 2380 Fälle; [6]). Aufgrund der SARS-CoV-2-Pandemie sind ansteigende Zahlen bei Anzeigen auf Verdacht einer BK (von 80.132 Fällen 2019 auf 106.491 Fälle 2020) zu erkennen, was einem Plus von 32,9 % entspricht; dies ist aber primär auf die BK 3101 „Infektionskrankheiten, wenn der Versicherte im Gesundheitsdienst, in der Wohlfahrtspflege oder in einem Laboratorium tätig oder durch eine andere Tätigkeit der Infektionsgefahr in ähnlichem Maße besonders ausgesetzt war“ zurückzuführen. Abzüglich der mit dem Coronavirus SARS-CoV‑2 in Zusammenhang stehenden Erkrankungen gingen die restlichen BK also leicht zurück. Dieser Rückgang könnte auch mit dem Rückgang der Zahl der ambulanten Behandlungsfälle im 1. Halbjahr 2020 (23 %) und der Veränderung der vertragsärztlichen Leistungsinanspruchnahme während der COVID-19-Krise assoziiert sein [7, 8].

Jährlich werden in Deutschland etwas mehr als 2000 Krebserkrankungen als Berufskrankheiten anerkannt [9, 10]. Zwei Drittel aller berufsbedingten Lungenkrebstodesfälle sind auf Asbest zurückzuführen [11]. 827 neue Erkrankungsfälle an Mesotheliom wurden 2019 in Deutschland als BK anerkannt, im Vergleich zu 2016 ein Rückgang (ca. 1000 Fälle; [11]). Berufsbedingte Plattenepithelkarzinome der Haut (BK 5103) sind mit 4023 Fällen nach Lärmschwerhörigkeit (BK 2301 mit 7414 Fällen) und Infektionskrankheiten (BK 3101 mit 18.969 Fällen) die dritthäufigste anerkannte Berufskrankheit im Jahr 2020 in Deutschland [10, 12, 13]. Damit stellen sie die häufigste anerkannte berufsbedingte Krebserkrankung dar.

Somit bleibt das Thema Berufskrankheiten, insbesondere maligner Erkrankungen, für das Gesundheitswesen insgesamt und vor allem für die Arbeitsmedizin hochrelevant. In der BK-Liste (Stand 2021) sind ein Viertel der Positionen Krebserkrankungen (17 durch toxische Stoffe, 2 durch physikalische Einwirkungen, wie u. a. ionisierende Strahlung und natürliche UV-Strahlung, sowie 1 durch biologische Einwirkungen, z. B. Hepatitis). Somit fällt das höchste Augenmerk auf die BK der Hauptgruppe 1 „Durch chemische Einwirkungen verursachte Krankheiten“ und ist diesbezüglich relevant für Fachärzte verschiedener Facharztrichtungen [13]. Lange Latenzzeiten zwischen Schädigungszeitpunkt und Erkrankungsbeginn, wie es u. a. bei der Asbestose bekannt ist [14, 15], erschweren den Entscheidungspfad für eine berufsbedingte Erkrankung. Somit kann in der BK-Statistik von einer erheblichen Dunkelziffer ausgegangen werden. Die frühzeitige Meldung einer BK ist vor allem für die Betroffenen hochrelevant, weil eine Verschlimmerung oder das Wiederaufleben der Erkrankung im Sinne der Individualprävention verhindert bzw. verzögert werden könnte [16]. Insbesondere onkologische Krankheiten führen häufig zu einer bedeutsamen Erwerbsminderung oder zum Tod des Versicherten.

Zwar wird davon ausgegangen, dass die Studierenden der Humanmedizin und Zahnmedizin Kenntnisse und Fähigkeiten zum Erkennen einer BK nach deutschem BK-Recht während des Studiums und die Ärzte während der Weiterbildung/Fortbildung erlernen, jedoch war die tatsächliche Situation bis dato kaum untersucht. Dieser Fragestellung widmete sich die hier vorgestellte Studie.

Ziel dieser Online-Befragung war es, die Ärzteschaft aus verschiedenen Fachgebieten in Bezug auf das Thema arbeitsbedingte Erkrankungen und Berufskrankheiten zu sensibilisieren. Es sollte der eigene Wissensstand zu Berufskrankheiten und das Handeln bei begründetem Verdacht auf eine BK selbst eingeschätzt werden. Zusätzlich dienten die Ergebnisse dieser Online-Befragung der Qualitätssicherung der arbeitsmedizinischen Lehre sowie Fort- und Weiterbildungen.

## Material und Methoden

### Probanden

Der Online-Fragebogen richtete sich an die Ärzteschaft verschiedener Fachrichtungen in Sachsen-Anhalt, unabhängig davon, ob eine Berufstätigkeit zum Erhebungszeitraum des Sommers und Herbsts 2014 vorlag oder nicht und wo das Medizinstudium abgeschlossen wurde. Der größte Anteil der Ärzteschaft kam aus dem niedergelassenen Bereich (87,4 %). Nur 8,3 % waren Klinikärzte, und der Rest verteilte sich auf Institute, Firmen, Ämter oder andere Einrichtungen.

### Methodik

Es wurde ein selbst konzipierter Fragebogen mit 28 Fragen genutzt. Dieser Fragebogen beinhaltete soziodemografische Daten, Daten zum Studienort, Weiterbildungsort, Facharztfachrichtung und Fragen zum selbst eingeschätzten Wissensstand über Zusammenhänge von beruflichen Expositionen und berufsbedingten Krebserkrankungen. Mehrfachnennungen waren teilweise möglich. Für die Beantwortung des Kenntnisstandes stand eine 6‑stufige Antwortskala im Sinne der klassischen Schulnoten sehr gut, gut, befriedigend, ausreichend, mangelhaft, ungenügend zur Verfügung. Offene Kommentare konnten ergänzt werden. Der Fragebogen war über die Website der Arbeitsgruppe erreichbar. Die Ärztekammer Sachsen-Anhalt und die Krankenhausgesellschaft Sachsen-Anhalt e. V. unterstützten die Befragung durch eine Sonderinformation an die niedergelassene Ärzteschaft und die entsprechenden Kliniken mit der Empfehlung einer Teilnahme an der Studie. Dies wurde durch einen E‑Mail-Newsletter übermittelt mit der Bitte um Weiterleitung an die verschiedenen Fachbereiche der Kliniken bzw. an die niedergelassene Ärzteschaft. Außerdem war die Befragung über die Website des Bereichs Arbeitsmedizin zu erreichen. Es wurde die Befragungs- und Prüfungssoftware EvaSys Qualitätsmanagementsystem (evasys GmbH, Lüneburg, Deutschland) genutzt.

Die Ethikkommission der Otto-von-Guericke-Universität Magdeburg gab ein positives Votum (Nr. 92/14) für diese Befragung, die im Zeitraum von 11/2014 bis 5/2015 erfolgt.

Die Daten werden rein deskriptiv dargestellt.

## Ergebnisse

### Soziodemographische Daten

Es nahmen 254 Ärzte, die in der Ärztekammer Sachsen-Anhalt registriert waren, an der Studie teil, wobei 58 % Frauen und 41,1 % Männer waren. 0,9 % machten keine Angabe zum Geschlecht. Es wurden Altersgruppen erfasst: 1,2 % der Studienteilnehmer waren unter 30 Jahre, 15,9 % 30–39 Jahre, 31,9 % 40–49 Jahre, 37,8 % 50–59 Jahre und 12,7 % über 60 Jahre alt. 0,4 % machten keine Angaben zum Alter. 61,4 % haben in Sachsen-Anhalt und 13,8 % in Sachsen studiert. Weitere Studienteilnehmer absolvierten das Studium in anderen Bundesländern (zwischen 0,4 und 5,9 %; bis auf Bremen, Rheinland-Pfalz, Brandenburg mit 0 %). Im Ausland studierten 5,5 % der befragten Ärzteschaft. 97,2 % der Stichprobe arbeiten in Sachsen-Anhalt. Von den Befragten waren 63,9 % Fachärzte, 20,2 % in leitenden Funktionen, 8,8 % Assistenzärzte, 4,6 % Chefärzte und 2,5 % Oberärzte. Die Stichprobe bestand aus 29,9 % Fachärzten für Allgemeinmedizin, gefolgt von 8,7 % für Innere Medizin. Der überwiegende Teil der Ärzteschaft (51,2 %) verfügte über 16 bis 30 Berufsjahre und 21,2 % über 30 bis 40 Berufsjahre.

### Kenntnisstand der Ärzteschaft zu Berufskrankheiten

**Figure 1:**
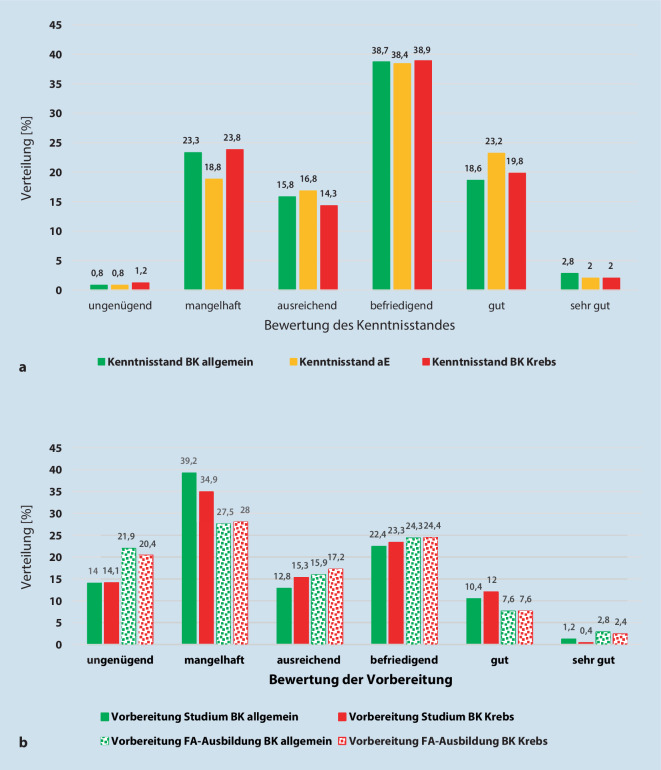

Nur ein Fünftel der Ärzteschaft gab zu dem Zeitpunkt der Befragung einen sehr guten bis guten selbsteingeschätzten Kenntnisstand zum Thema BK allgemein an. Knapp 40 % äußerten befriedigende, 15,8 % ausreichende und 23,3 % mangelhafte Kenntnisse in den 3 Bereichen. Ein sehr geringer Prozentsatz (0,8 %) der Befragten verfügte nur über ungenügende Kenntnisse in allen 3 Bereichen. Die Daten sind in Abb. 1a dargestellt.

Geringfügig besser fiel die Frage zum Kenntnisstand über die arbeitsbedingten Erkrankungen aus. Hier schätzte jeder vierte Arzt seinen Kenntnisstand gut oder sehr gut ein. Nur noch 23,3 % bewerteten ihre Kenntnisse als mangelhaft. Der überwiegende Teil der Befragten (28,6–71,4 %) schätzten befriedigende Kenntnisse bei den Themen BK allgemein.

**Figure 2:**
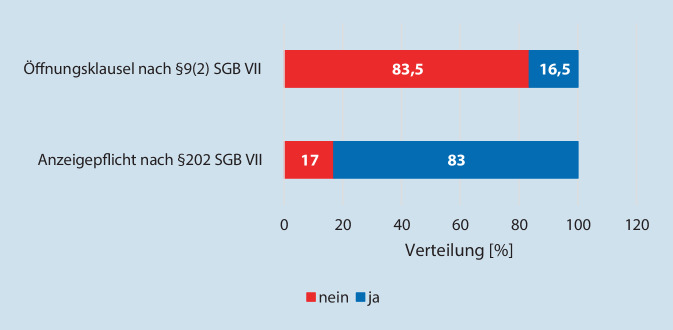

Insgesamt 83 % aller Befragten kannten die Anzeigepflicht nach § 202 SGB VII bei einem begründeten Verdacht auf eine Berufskrankheit. Bei den Absolventen des Medizinstudiums im Ausland war es nur jedem zweiten bekannt. Die Ergebnisse der Befragung zeigten des Weiteren, dass die Öffnungsklausel nach § 9 Abs. 2 SGB VII nur 16,5 % der Ärzte bekannt war. Die Ergebnisse sind grafisch in Abb. 2 dargestellt.

### Vorbereitung auf das Thema Berufskrankheiten im Studium und während der Facharztausbildung

Bei der Suche nach einem Verbesserungspotenzial im Kenntnisstand zu dieser Thematik wurden Fragen zur Vorbereitung auf das Thema Berufskrankheiten im Studium, in der Facharztausbildung und bei den Fort‑/Weiterbildungsveranstaltungen gestellt.

Die Darstellung der Ergebnisse ist in Abb. 1b aufgezeigt. Sie zeigten, dass nur etwa 12 % eine sehr gute oder gute und etwa 22 % eine befriedigende Vorbereitung zum Thema Berufskrankheiten im Studium oder während der Facharztausbildung (FA) erlangten. Bei mehr als der Hälfte war sie nur ausreichend (12,8 %), mangelhaft (39,2 %) oder ungenügend (14 %). Die Daten für das Thema berufsbedingter Krebs sind vergleichbar.

Im Rahmen der Facharztausbildung wurde das Thema Berufskrankheiten ebenfalls abgefragt, wobei 10,4 % der Befragten es als sehr gut oder gut, 24,3 % als befriedigend, 15,9 % als ausreichend, 27,5 % mangelhaft und 21,9 % als ungenügend bewerteten. Vergleichbar verhielt es sich in der Facharztausbildung bei der Frage zum Thema berufsbedingte Krebserkrankungen.

Retrospektiv unterschätzen jeweils 46,9 % der Ärzte teilweise und 46,9 % ganz die Relevanz des Themas BK allgemein während des Studiums. Nur 6,1 % der Ärzte meinen, dass aus ihrer heutigen Sicht, die Thematik Berufskrankheiten allgemein während des Studiums nicht deutlich unterschätzt wird. Die Daten für das Thema BK Krebs liegen ähnlich (50 % teilweise, 41,8 % ganz).

### Wahrnehmung von Fortbildungen zum Thema Berufskrankheiten

**Figure 3:**
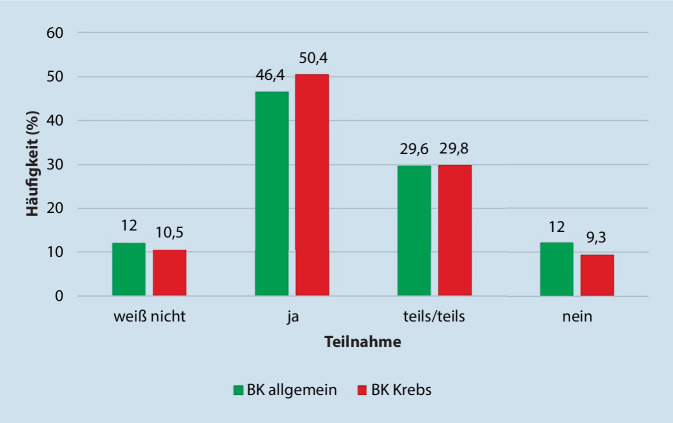

Die deutliche Mehrheit der Ärzteschaft nahm Fortbildungsangebote zu dem Thema Berufskrankheiten allgemein (72,2 %) oder BK Krebs (78,1 %) nicht wahr. Die Mehrheit würde jedoch zu beiden Themen an Fortbildungsveranstaltungen teilnehmen (Abb. 3).

### Angezeigte Verdachtsfälle und deren Verlauf

Alle Ärzte haben in ihrer ärztlichen Tätigkeit bereits eigenständig eine Anzeige beim Verdacht auf eine Berufskrankheit erstellt; 86,3 % der Ärzteschaft weniger als 25 Verdachtsfällen auf eine BK angezeigt. 7,7 % taten es bei 26–50 Fällen, 4 % bei 51–100 Fällen und 2 % bei über 100 Fällen, wobei diese Statistik nicht nach Altersgruppen bereinigt ist. Von diesen angezeigten Fällen war das Ergebnis der Begutachtung der Berufskrankheiten (alle), wie viele der eigenständig angezeigten Verdachtsfälle anerkannt wurden, zu 75 % und bei den berufsbedingten Krebserkrankungen zu 79,7 % den Ärzten nicht bekannt.

### Offene Kommentare und Kritikpunkte der Ärzteschaft

Nachfolgend werden auszugsweise Kommentare der Ärzteschaft aufgeführt, die zur Diskussion beitragen können (ohne Berücksichtigung der Kommentare hinsichtlich des Wissenstests):

- „… Wichtig, die Inhalte müssen unbedingt in das Studium aufgenommen werden und unbedingt in die Krankenhäuser, wir vermissen die Berufsanamnese in den meisten Fällen …“
- „Der Ablauf der Verdachtsmeldung einer Berufserkrankung sollte bei Weiterbildungen mit gelehrt evtl. umwelt- und berufliche Noxen erörtert und den Erkrankungen zugeordnet als Liste zur Verfügung gestellt werden. KV- oder ÄK-Zuarbeiten wären hilfreich für Ärzte in Selbstständigkeit …“
- „Es ist erschreckend wie wenig das Thema bisher mein Interesse weckte. Danke für die Anregung.“
- „Ich würde mich freuen, wenn mir eine Weiterbildung zu Berufskrankheiten allgemein und auch zu Berufskrankheiten und Krebserkrankung ‚über den Weg läuft‘ und ich würde gerne an einer teilnehmen.“
- „In meiner Facharztausbildung zum FA für Allgemeinmedizin (1975–1981) gehörte eine Weiterbildung im Fach Arbeitsmedizin dazu – was ich für unbedingt nötig halte, auch unter heutigen Bedingungen!“
- „Sicher wurden im Studium schon die wesentlichen berufsbedingten Erkrankungen behandelt. Vieles ist aber in Vergessenheit geraten, wenn man nicht ausgesprochen arbeitsmedizinisch tätig ist …“
- „Wenn ich die Patienten an einen Facharzt überweise, gehe ich davon aus, dass die Meldung nach Sicherung der Diagnose von dort erfolgt. Außerdem ist m. E. der Fragebogen/die Hürde für die Anerkennung der BK sehr aufwendig/hoch.“
- „Die Weiterbildung steht und fällt mit dem Einsatz des Ausbilders, die Zeit dieser Ausbildung muss einem solchen Arzt zugestanden werden, im Klinikalltag kommt diese Zeit häufig zu kurz, viele Ausbilder sind leider keine guten Lehrer.“
- „… Die Einschätzung einer möglichen BK ist oft kaum möglich, da die Arbeitnehmer über die toxischen Substanzen kaum informiert sind und oft Chemikalien (Beipackzettel o. ä.) aus Betriebsschutzgründen nicht beigebracht werden können. Im Übrigen haben wir auch einfach zu wenig Kenntnisse über die Arbeitsplätze in der Region. Daher halte ich regionale Informationsveranstaltungen und Arbeitsplatzdarstellungen für angebracht.“
- „Welche kanzerogene Stoffe in welchen Betriebszweigen verwendet werden, ist in der Praxis meist nicht bekannt … auch die Arbeitnehmer wissen darüber nicht Bescheid!!“
- „Über Berufs-Erkrankungen ist viel zu wenig bekannt.“

## Diskussion

Die Online-Befragung der Ärzteschaft zum Thema Kenntnisse über BK allgemein oder BK Krebs und deren Vorbereitung im Studium und FA-Weiterbildung war eher ernüchternd. Die befragte Ärzteschaft war überwiegend regional in Sachsen-Anhalt verteilt; eine Übertragung auf andere Bundesländer ist jedoch denkbar. Die Altersverteilung spiegelt den Altersdurchschnitt der Ärzteschaft durchaus wider. So ist das Durchschnittsalter der Ärzte und Psychotherapeuten in der vertragsärztlichen Versorgung zwischen 2011 und 2020 von 52,7 auf 54,2 Jahre angestiegen [17]. Der Kenntnisstand zu Berufskrankheiten ist trotz der vielen Berufsjahre als befriedigend bis mangelhaft einzuschätzen. Die Befragten konnten nicht ausreichend während des Studiums oder während der Facharztausbildung zu dieser Thematik vorbereitet werden oder nahmen keine Fortbildungsveranstaltungen zu BK während der Praxis‑/Kliniktätigkeit wahr. Insgesamt wurde die Relevanz der arbeitsmedizinischen Thematik bereits während des Studiums unterschätzt, Defizite konnten auch nicht in der Facharztausbildung oder während der Berufstätigkeit verbessert werden. Für Ärzte, die im Ausland studierten und die Approbation in Deutschland anerkennen lassen wollen, bedeutet das, dass diese Ärzte über Kenntnisse zum deutschen Berufskrankheitenrecht verfügen müssen, jedoch diese Voraussetzungen nicht geprüft werden [18]. Eine Prüfung hierzu erscheint sinnvoll, da sich die rechtlichen Grundlagen zu dieser Thematik innerhalb der EU unterscheiden. So ist bspw. die europäische Liste der Berufskrankheiten keine gesetzlich verbindliche Regelung und dient nur als Empfehlung mit präventiver Zielrichtung [19]. Dem Anforderungskatalog an ausländische Ärzte für die Anerkennung der Approbation in Deutschland ist nicht zu entnehmen, dass in der BRD eine Verpflichtung zur Meldung über BK sowie die damit verbundene erforderliche Kenntnis über die Diagnose von BK besteht [18]. Dieser Kenntnislücke könnten ggf. verpflichtende arbeitsmedizinische Kurse verschiedener Institutionen entgegenwirken. Diese würden den hohen fachlichen Anforderungen an die in Deutschland arbeitende Ärzteschaft fördern.

Als limitierender Aspekt der Studie ist die viel zu niedrige Beteiligungsquote der befragten Ärzteschaft von 2 % anzusehen, sodass die Erkenntnis nicht verallgemeinert werden kann. Allerdings erweckt die niedrige Teilnahme der anhaltinischen Ärzteschaft den Eindruck, dass zum Erhebungszeitpunkt allgemein geringes Interesse an den Online-Befragungen oder an der Arbeitsmedizin bestand. Die Daten sind aus dem Jahr 2014, also vor der Einführung des Nationalen Kompetenzbasierten Lernzielkatalogs Medizin (NKLM) 2.0. Interessant wäre sicherlich auch der Vergleich zum Status quo nach Einführung und Etablierung des NKLM. Eine Facharzt-Konfundierung wäre interessant gewesen, konnte aber wegen kleiner Fallzahlen in den einzelnen Facharztgruppen nicht durchgeführt werden.

Ein Grund für die Unterschätzung der Thematik ist vermutlich der späte Einzug der Arbeitsmedizin in das Studium; möglicherweise hat sich schon ein Großteil der Studierenden für eine spätere Fachrichtung entschieden und interessierte sich weniger für das arbeitsmedizinische Fach. Wahlpflichtfächer, die bereits ab dem 3. Studienjahr angeboten werden, könnten hier teilweise Abhilfe schaffen. Es sollte versucht werden, arbeitsmedizinische Inhalte schon frühzeitig im Studium zu implementieren. Denkbar wäre ein POL-Unterricht in der Arbeitsmedizin als Wahlpflichtfach oder die fakultative Lehre. Des Weiteren unterstützt der Beitrag und die Fachempfehlung Arbeitsmedizin im NKLM 2.0 die Bedeutung des Faches im Studium [20]. Hierbei könnten Fallbeispiele helfen, die die Relevanz der Arbeitsanamnese üben und bei denen diskutiert wird, ob eine BK-Verdachtsdiagnose zur Anzeige gebracht werden sollte und wie dies dann abläuft. Denkbar ist auch ein interdisziplinärer Unterricht mit Fachdisziplinen, bei denen Berufskrankheiten häufiger vorkommen. Auch hier wäre eine Fallkonstellation von Anamneseerhebung bis Anzeige einer BK denkbar. Die arbeitsmedizinischen Kollegen könnte man zu Fallkonferenzen in den Kliniken einladen. Die Bestrebungen, das Thema BK besser in der Lehre zu verankern, sind schon jetzt in dem NKLM 2.0 ersichtlich.

Problematischer erscheint die Erreichbarkeit der Ärzteschaft bei der Weiterbildung in den Krankenhäusern. Hier wären Fortbildungsmodule durch die jeweiligen Ärztekammern zu empfehlen. Fachärzte würden hiervon auch profitieren. Die Schaffung von Newslettern zur Verteilung von Fortbildungsmaterialien ist ebenfalls denkbar.

## Fazit für die Praxis

- Die Arbeitsmedizin ist eine spannende, interdisziplinär vernetzte Fachdisziplin, deren Relevanz deutlich von Studierenden der Medizin unterschätzt wird.
- Die frühe Präsenz der Arbeitsmedizin und eine interdisziplinäre Zusammenarbeit mit anderen Fachdisziplinen im Studium sollten angestrebt werden.
- Die Kommunikation und der Aufbau interdisziplinärer Fallbesprechungen und Klinikkonferenzen sollten unter Berücksichtigung der Arbeitsanamnese erfolgen.
- Interdisziplinäre Fortbildungsangebote mit den Arbeitsmedizinern sollten regelmäßig bei den zuständigen Ärztekammern angeboten werden.

## Footnotes

1 Aus Gründen der leichteren Lesbarkeit wird hier u. a. die männliche Sprachform bei personenbezogenen Substantiven und Pronomen verwendet. Dies impliziert jedoch keine Benachteiligung der weiteren Geschlechter, sondern soll im Sinne der sprachlichen Vereinfachung als geschlechtsneutral zu verstehen sein.
